# Increased School Breakfast Participation from Policy and Program Innovation: The Community Eligibility Provision and Breakfast after the Bell

**DOI:** 10.3390/nu14030511

**Published:** 2022-01-25

**Authors:** Dan Ferris, Jason Jabbari, Yung Chun, J.S. Onésimo Sándoval

**Affiliations:** 1 Social Policy Institute, Washington University in St. Louis, Saint Louis, MO 63130, USA; jason.jabbari@wustl.edu (J.J.); yungchun@wustl.edu (Y.C.); 2 College of Arts and Sciences, Saint Louis University, Saint Louis, MO 63108, USA; ness.sandoval@slu.edu

**Keywords:** child nutrition, school meals, school breakfast programs, policy, food insecurity, nutrient sources

## Abstract

School meals provide significant access to food and nutrition for children and adolescents, particularly through universal free meal mechanisms. Alongside added nutritional meal requirements under the Healthy, Hunger-Free Kids Act (2010), schools can utilize meal program and policy mechanisms such as the Community Eligibility Provision (CEP) and Breakfast after the Bell (BATB) to increase participation. This study examines longitudinal statewide school-level CEP and BATB adoption and estimates the impact on increased free and reduced-price (FRP) breakfast participation. We find that FRP breakfast participation increased for schools that utilize both CEP and BATB (14-percentage-point increase) and that CEP-participating schools are more likely to use BATB approaches such as breakfast in the classroom, grab-and-go carts, and second-chance breakfast. Additionally, using a conditional Difference-in-Differences (DiD) approach, we find that BATB adoption accounted for a 1.4-percentage-point increase in FRP school breakfasts served (*p* < 0.05). Study findings can inform policy and school official decision making around the policy and program mechanisms at their disposal to increase school meal participation and student nutrition.

## 1. Introduction

Numerous policies and programs aim to support childhood nutrition and dietary intake in the United States (U.S.), particularly for families who experience difficulty consistently accessing healthy, nutrient-rich meals. Distinct from malnourishment or hunger, which represent physiological conditions or physical states resulting from inadequate intake of nutrients, food security uses a broader lens of the economic and social factors that impact households with wide-ranging immediate, short-, and long-term consequences. Considered the lack of “access by all people at all times to enough food for an active, healthy life”, 10.5% of all U.S. households and 14.8% of households with children experienced food insecurity in 2020 [1].

Research has shown that food insecurity negatively affects children’s health [2], development [3], and academic performance [4]. Research has also shown the critical role of school meals for youth and adolescent nutrition, dietary intake, and health. When considering the long-established connection between poverty status and food insecurity [5], as well as the negative effects of food insecurity on child development [6], unsurprisingly, school meal programs have been found to reduce food insecurity [7,8] while improving health [9] and education outcomes [10,11,12]. Pertaining to child nutrition and dietary intake, children participating in school meals in the U.S. have a lower prevalence of inadequate nutrients [13], which is especially important when considering that meals brought from outside of school have been found to be of lower nutritional quality than meals received inside school [14,15]. One study that used 24-hour dietary recall found that school breakfast and lunch made up nearly half of the total calories consumed by students, including notable amounts of vegetables and dairy [16]. Notably, implementation of school food environment, policy, and practice changes can lead to improved student diets with the potential to reduce obesity [17,18]. Universal school breakfast, specifically, increases the likelihood that elementary school students eat a nutritionally substantive breakfast [19].

A comprehensive view of food insecurity and opportunities for effective interventions can also be viewed using a cyclical food insecurity and chronic disease framework [20]. Here, chronic disease incidence leads to increased food insecurity, and increased food insecurity has been found to complicate and exacerbate chronic disease conditions. Key to the cycle, food insecurity leads to increased stress for meeting basic household needs and chronic disease incidence, which in turn contributes to increased health care expenditures, potential for reduced employment, decreased income, and increased spending tradeoffs. These in turn amplify and extend household food insecurity. Therefore, increased access to FRP school meals can have far-reaching implications not only for students but also for households [7,8].

In addition to the Supplemental Nutrition Assistance Program (SNAP) and the Special Supplemental Nutrition Program for Women, Infants, and Children (WIC), the National School Lunch Program (NSLP) and School Breakfast Program (SBP) have a particularly expansive reach and impact on child nutrition and meal access. School meals under the SBP and NSLP must meet specific nutrition criteria established by the United States Department of Agriculture (USDA) in order to receive federal subsidies and reimbursement and serve as many as 30 million children lunch and 15 million children breakfast each day [1,21]. These programs provide an especially critical source of nutrition for the estimated 6.1 million children living in food-insecure households and 584,000 children in households with children experiencing very low food security [1]. Notably, 76.9% of school lunches and 87.7% of school breakfasts were served to free or reduced-price eligible students in fiscal year 2020 [22]. School meals have also been found to contribute significantly more to energy intake for food-insecure and marginally food-secure students [23].

Despite the significant benefits of school meals, however, a substantial number of eligible students do not participate in free and reduced-price (FRP) school meals, with school breakfast in particular historically lagging behind school lunch [7]. While school breakfast has come a long way since its formal introduction in 1966 and first known national evaluation in 1971 in which only eight percent of the schools participating in the NSLP had started to serve breakfast [24], the continuing gap between breakfast and lunch warrants policy and program level interventions to increase participation in addition to various school cafeteria practices.

Obstacles for increasing student participation include stigma [25] and repercussions for unpaid meal debt [26]. Students lack the time or desire to eat school breakfast and report that school breakfast foods do not align with their taste preferences [27,28]. How schools administer meal programs has significant implications for student participation as well, as schools face a bevy of meal program administrative and logistical considerations [29]. In addition to food quality and the location and environment in which meals are available, the time allocated to eat can affect student decision making about school meals [30,31]. Nationally, nearly half of school districts do not require or recommend schools provide students with at least 20 minutes of “seat time” to eat their meal [32]. A recent systematic review outlines key practices that schools can adopt, which include recipe adaptation, incentivizing fresh food consumption, and increased menu choices [33].

To reduce some obstacles to increasing access to healthy school meals, the Healthy Hunger-Free Kids Act of 2010 (HHFKA) was established. Among other policy provisions, the HHFKA created CEP for improved school meal administrative efficiency, as well as effectiveness in increasing student participation through universal free meal access with school incentives for higher reimbursement rates. For a family of four, free school breakfast and lunch are available to students with household incomes below 130% of the Federal Poverty Level of USD 26,500 in 2021. Students with household incomes below 185% of the Federal Policy Level qualify for reduced-price meals. Schools that participate in CEP determine student eligibility for free meals at the school level rather than through individual family applications. Specifically, CEP eligibility is determined by an Identified Student Percentage (ISP)—the direct certification of the number of students receiving other federal programs such as SNAP, Medicaid, or Temporary Assistance for Needy Families (TANF), or who have an identified status of being homeless or in foster care. Schools can participate in CEP at and above 40% ISP and receive the highest reimbursement rate for free meals served when the ISP reaches 62.5%. Research on the early impacts of HHFKA and CEP suggests gradually increased meal participation rates following its passage [34] and significantly improved nutrition standards [35]. At the same time, universal school meals have positive associations with diet quality, food security, and academic performance, as detailed in a recent systematic literature review [10].

Prior to HHFKA and CEP, significant efforts toward innovating school meal service delivery centered on increasing breakfast participation through Breakfast after the Bell (BATB) approaches. One approach, breakfast in the classroom, intends to mitigate time constraints and reduce stigma [36,37]. Similarly, second-chance breakfast and grab-and-go carts support students who otherwise would miss breakfast, feel hungry later in the morning, or have time constraints with traditional breakfast cafeteria service models [38,39]. These BATB approaches either supplement or replace the traditional before-school cafeteria-style breakfast to increase access and participation. Research has demonstrated increased participation from second-chance breakfast [38], breakfast in the classroom [31], and grab-and-go [40], as well as improved student achievement [41] and attendance [42]. Schools vary in their approaches to school breakfast, using some, none, or all of these methods in addition to or instead of traditional cafeteria-style service.

While extensive research has examined the impacts of CEP or BATB approaches, our study examines the relationship between CEP and BATB with school breakfast participation. To increase understanding of policy and program mechanisms for school breakfast service, we utilize both descriptive and inferential methods. Using Missouri statewide longitudinal school administrative data, this study explores how BATB and CEP adoption together and separately relate to changes in FRP breakfast participation over time. Then, using a conditional Difference-in-Differences (DiD) strategy, we employ propensity score matching techniques to estimate the impact of program and policy adoption on FRP breakfast participation.

We find that CEP-participating schools are more likely to also use BATB approaches and that both are related to increased FRP breakfast participation. BATB adoption in particular accounts for a statistically significant 1.4-percentage-point increase in FRP school breakfasts served. We discuss these findings in the context of CEP and BATB’s wide-ranging elements and outcomes and conclude that school-level stakeholders and policy decision makers may have the greatest impact on increasing breakfast participation by taking a multipronged policy and program approach.

## 2. Materials and Methods

### 2.1. Data and Measures

This study explores the association among CEP, BATB, and FRP breakfast provision in Missouri schools between September 2016 and March 2020. Data for our analysis come from four school-level datasets provided by the Missouri Department of Elementary and Secondary Education (DESE). A demographics dataset provides annual (school year) demographic information of enrolled students at each Missouri school. The Community Eligibility Provision (CEP) dataset includes eligibility for and participation in the CEP program at the school level over the study period, including school ISP, the percentage of students automatically certified for free meals due to their participation in federal benefit or assistance programs such as SNAP, TANF, and Head Start. The program dataset provides detailed information on school meal program participation. The meal service dataset provides the number of full-price, reduced-price, and free breakfasts and lunches served for each school. In addition, the meal service data provides the number of students who are eligible for FRP meals, number of meal days, and average daily attendance. 

To merge the meal service dataset with demographic and CEP data, we collapsed monthly information into yearly information (excluding May through August, when summer break often occurs). We focus on traditional cafeteria-served breakfast and Breakfast after the Bell (BATB), which includes breakfast in the classroom, grab-and-go carts, and second-chance breakfast. As CEP eligibility information is missing for some schools in certain years, we impute missing values using interpolation techniques [43]. To account for observed missing and abnormal data such as schools with substantially more meal days reported in a month, we limit our final sample to remove outliers and the most peripheral 1% of schools reporting FRP breakfasts served.

### 2.2. Analysis and Estimation Models

In order to understand BATB, we utilized both descriptive and inferential methods. When considering the large number of Missouri students who are eligible for but do not receive free and reduced-price breakfasts, we explored the relationship between school CEP and BATB adoption, as well as whether CEP and BATB implemented together, increases meal participation. We first demonstrate changes in BATB, CEP, and FRP breakfast participation over time. Next, we explore the relationship between CEP and BATB. Finally, we interact CEP and BATB, providing marginal effects on FRP breakfast participation.

Concerning the impact of both CEP and BATB on FRP breakfasts in Missouri, it is important to note that adoption is staggered, and the duration of schools utilizing CEP or BATB varies. Traditional DiD approaches, which are used to estimate policy impacts across adopters and non-adopters over time, are often limited in their ability to deal with staggered and time-varying treatments. This can be especially problematic when treatment effects vary over time. Moreover, traditional DiD approaches often assume similar pre-treatment trends and are thus limited in their ability to deal with policy adopters and non-adopters who differ on both observed and unobserved characteristics. 

We therefore use a conditional DiD approach that uses propensity scores and coarsened exact matching to select treatment and control groups that are similar on observed characteristics. Flexpaneldid is a flexible-panel DiD approach that consists of an open-source toolbox developed in STATA [44]. Here, each treatment unit is assigned a set of similar control units observed at the same time. In doing so, the conditional DiD approach uses a weighted average of differences across treatment and control units to estimate treatment effects. While this approach can decrease selection bias and increase internal validity, this approach can also limit the total sample size and threaten external validity. Nevertheless, as our main interest is understanding the impact of BATB on meal participation, a conditional, rather than canonical, approach is most appropriate. A mathematical representation and specification of our conditional DiD model is:

(1) $$ATT=\frac{1}{I}\overset{I}{\underset{i=1}{\sum }}\left[\left(Y_{i,t_{0i}+\beta_{i}}-Y_{i,t_{0i}}\right)-\left(Y_{j,t_{0j}+\beta_{i}}-Y_{j,t_{0i}}\right)\right]$$

where I is the number of matched pairs, whereas i and j are treated schools and their respective matched controls, respectively; $t_{0i}$ is individual treatment start dates (i.e., BATB start year); $t_{0i}+\beta_{i}$ reflects the individual duration from treatment start to outcome observation; and Y is the number representing FRP breakfast provision.

In our conditional DiD model, coarsened exact matching was used to match treated schools to schools that were identical in terms of CEP eligibility, urbanicity (metropolitan area vs non-metropolitan area), food access, region (identified by DESE), school type (i.e., public, private, charter), and grade level (i.e., elementary, middle, and high). Our measure of food access was provided by the USDA and defines low access as “a tract with at least 500 people (or 33% of the population) living more than 1 mile (urban areas) or 10 miles (rural areas) from the nearest supermarket, supercenter, or large grocery store” [45]. Propensity scores were used to select similarly sized schools with similar sizes (school enrollment) and demographic information (percent of students identifying as White). Propensity scores also matched treatment and control schools on pretreatment outcomes in the year prior to the treatment start date. Our results demonstrate the impact of BATB adoption after one year.

## 3. Results

Our descriptive analysis indicates that CEP participation has gradually increased over the study period. In the 2016–17 academic year, 42.3% of CEP-eligible schools participated in the CEP program. As of the 2019–2020 academic year, 60.4% of eligible schools participated in CEP, which represents 38.1% of all Missouri schools identified that year as eligible, nearly eligible, or ineligible in state data (assuming missing schools are neither eligible nor participating brings the percent of all Missouri schools participating overall notably lower). BATB participation also increased over the study period. First collected in Missouri in 2016 and systematically in 2017, BATB adoption and implementation data indicate that the schools utilizing a BATB approach increased from 36.3% (2017–2018) to 46.3% (2019–2020) in a three-year period. By the 2019–2020 academic year, almost one thousand Missouri elementary, middle, and high schools (*n* = 981) utilized at least one BATB method. It is also important to note that schools increasingly have provided students with multiple BATB options (146 schools used more than one BATB approach in 2017–18, which increased to 275 schools in 2019–2020). FRP breakfast participation increases over a similar time period, however more subtly (48.8% in 2016–2017 to 51.1% in 2019–2020). Table 1 reports descriptive summary statistics of the key indicators over the study period and covariates for the analytic models.

**Figure 1:**
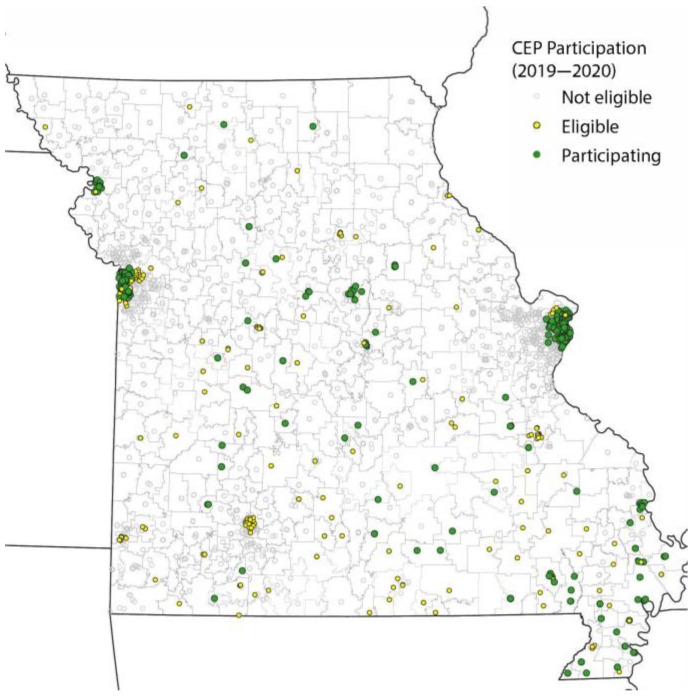
School CEP status (2019–2020).

Geographically, while there is at least some level of school CEP participation in all Missouri regions, we see that CEP-eligible and participating schools are highly concentrated in urban areas, including St. Louis City and Kansas City (Figure 1).

**Figure 2:**
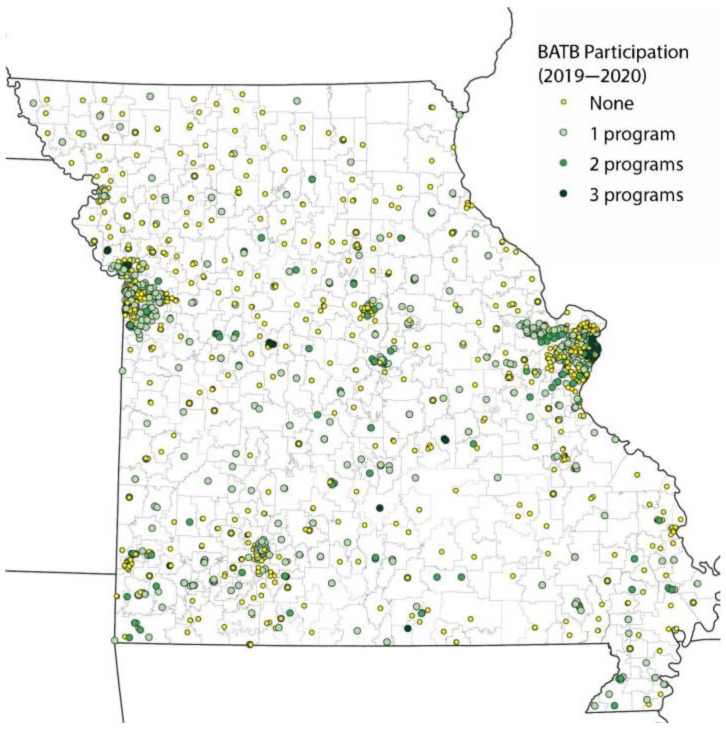
School BATB adoption (2019–2020).

Similarly, BATB participation was highly concentrated in urban areas, such as St. Louis City (Figure 2).

**Figure 3:**
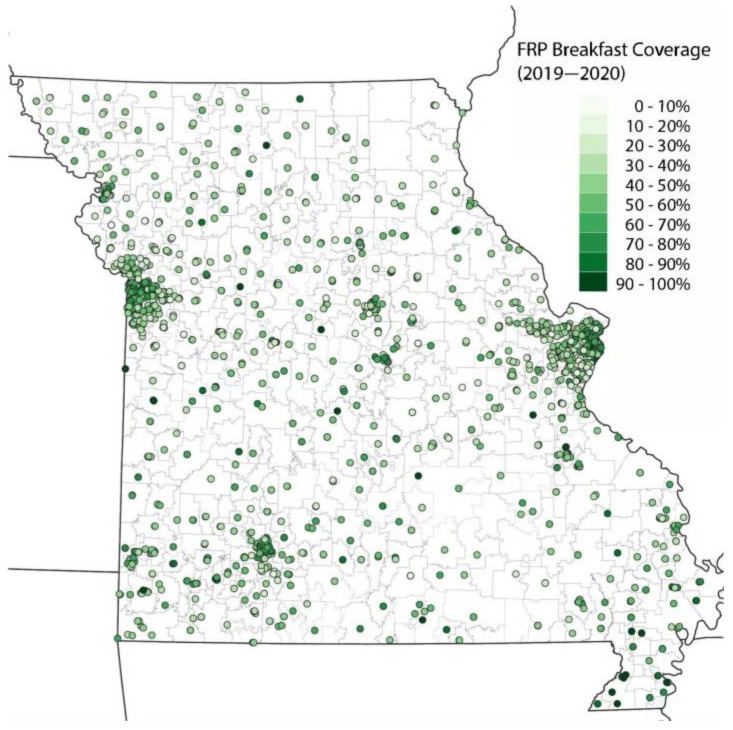
School FRP breakfast coverage (2019–2020).

While FRP breakfast did not show strong spatial patterns, it is still notable that schools with high FRP breakfast coverage were concentrated in urban centers (Figure 3).

**Figure 4:**
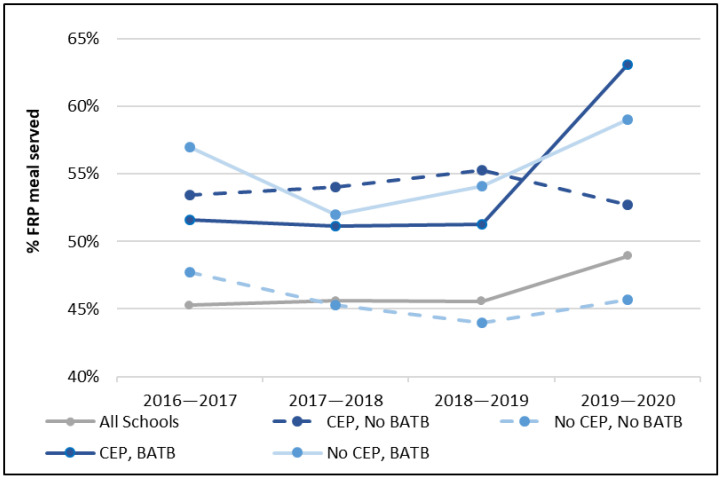
Annual change in breakfast program participation.

We then examined annual changes in breakfast participation for schools with different combinations of CEP and BATB adoption (Figure 4). Here, we see that while BATB adopting schools are associated with large increases in free and reduced-price breakfast participation, these increases are largest for schools that also adopt CEP and most prominently in the 2019–2020 school year. Data for BATB policy adoption begin in the 2017–2018 school year and are used to demonstrate 2016–2017 patterns for schools that adopted BATB the following year.

Combining data across years, we find that schools that participated in CEP had 94% greater odds of utilizing one or more BATB approaches. As school characteristics can heavily influence CEP eligibility and participation, we limited our sample to only CEP-eligible schools, and the significant results were maintained, demonstrating the strength of CEP in its association with BATB. Here, CEP could provide the flexibility or incentives for schools to try more innovative methods in serving meals to students.

**Figure 5:**
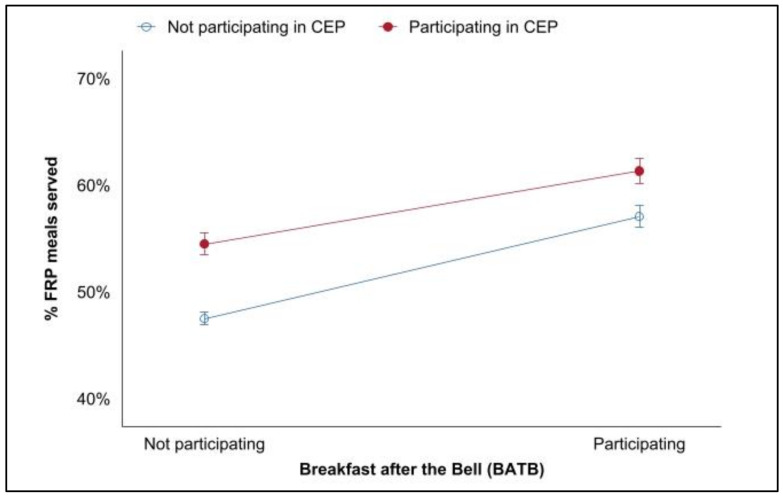
Impacts of CEP and BATB on FRP breakfast provision.

We also found that while both CEP and BATB have their own positive association with the proportion of free and reduced-price breakfasts served, schools that utilize both CEP and BATB together serve substantially more FRP breakfasts (Figure 5). Schools participating in only CEP were associated with a 7-percentage-point increase in the proportion of FRP breakfasts served; schools participating in only BATB were associated with a 10-percentage-point increase, and participating in both CEP and BATB were associated with a 14-percentage-point increase when compared to schools that participated in neither.

Finally, we use a conditional (DiD) model to examine the impact of schools adopting BATB on the proportion of FRP breakfasts served the following year (Table 2). When compared to similar non-adopting schools, we found that schools adopting BATB experienced a statistically significant 1.4-percentage-point increase (*p* < 0.05) in the proportion of free and reduced-price breakfasts served, a total of roughly 756,000 more breakfasts served per year.

To examine the robustness of our conditional DiD findings, we examined mean treatment effects using a canonical (traditional) DiD model across academic years 2017–2018 through 2019–2020 (Table 3). These findings confirm our conditional DiD model.

## 4. Discussion

Despite the demonstrated benefits of school meals, a substantial number of FRP-eligible students do not partake, especially in school breakfast. With significant barriers for students accessing school breakfast through traditional meal service mechanisms, BATB approaches provide schools with additional options to increase access and participation. Nevertheless, there is little research that has looked longitudinally at statewide BATB adoption and considered meals served to those who can most benefit—FRP-eligible students. Using both descriptive and inferential approaches, our study finds that CEP is associated with BATB adoption and that both are related to increased FRP breakfast participation. From our conditional DiD analysis, we find that BATB adoption alone accounts for a 1.4-percentage-point increase in school breakfasts served. These findings build on previous studies that looked independently at CEP or BATB, and an increasing amount of research that uses longitudinal approaches (e.g., DiD) alongside more traditional descriptive analyses.

While still a relatively novel policy option, CEP adoption suggests it eases administrative burdens and incentivizes innovative methods of meal service delivery as seen through increased BATB implementation in CEP-participating schools. As highlighted by Hearst [46], however, program implementation does not inherently lead to changed student perception or sustained change. While BATB and CEP can help to address stigma and accessibility of meals, food-insecure students may still not participate if foods do not meet their preferences and needs. As Cohen et al. find in their recent systematic review, key practices schools can adopt include recipe adaptation, incentivizing fresh food consumption, and increased menu choices [33].

Additionally, while our conditional DiD methods account for geographic differences in food access, one notable recent study of Missouri schools points out that because of the 40% ISP threshold, schools adopting CEP likely already have large percentages of FRP-eligible students, an estimated 79% [47]. Therefore, CEP by itself may have limited impacts on increasing FRP meal participation, which aligns with our descriptive results. Practically, BATB models also can present obstacles for schools and meal service departments, including implementation costs, reliance on additional staff and teachers outside the cafeteria for successful service delivery, and additional communication with parents. Both the administrative and programmatic opportunities and potential challenges for increasing school meal access present significant considerations for schools in Missouri, the U.S., and beyond for increasing student meal participation and nutrition.

Significant school meal program research to date includes a descriptive analysis of either CEP or BATB meal service approaches in relation to the numbers of meals served, oftentimes looking at an approach in isolation of the larger range of mechanisms schools have at their disposal to increase free meal and breakfast participation. Our study’s greatest strengths and unique contribution rest with its use of statewide, longitudinal data examining associations for both CEP and BATB with FRP breakfast participation. Additionally, we use a conditional DiD approach, which accounts for a variety of potential confounders while balancing treatment and control schools of pre-treatment outcomes. The conditional DiD approach also accounts for selection bias and strengthens the internal validity of the study. This methodology expands upon recent work that used a multiple logistic regression approach and statewide cross-sectional data in North Carolina from October 2017 to examine SBP participation in relation to both CEP and BATB, finding positive associations between free school breakfast participation independent of and in combination with BATB [48]. Other previous studies have used DiD approaches to examine CEP among elementary school students [49] in relation to education outcomes [12,50] and regarding weight status [51].

Nevertheless, our study is not without limitations. Employing a conditional DiD approach uses a smaller sample of comparable schools, which can potentially limit external validity. As our analytic sample still has roughly half the number of total Missouri schools, we believe that issues of external validity are less prominent in our main analysis. In addition, our approach does not distinguish between BATB methods (e.g., breakfast in the classroom, second-chance, or grab-and-go) in order to preserve the treatment size. Each approach in isolation or combination may not have the same impact on participation. As schools make decisions on innovative breakfast programs, future research should further explore the differences between types of BATB programs. 

While we control for school level, type, size, and other factors in isolating the effects of BATB and CEP, our study also does not distinguish between associations or effects specifically at the elementary, middle, or high school levels, which can also guide stakeholder decisions. Finally, while the statewide approach extends beyond a number of previous studies that looked exclusively at rural or urban schools, we acknowledge generalizability considerations from matching schools in the policy context of one state and look forward to the continued advancement of longitudinal analysis of the implementation of BATB and CEP elsewhere and, ultimately, across multiple states.

## 5. Conclusions

CEP and BATB present opportunities for schools to increase student free meal eligibility, as well as participation. Our findings demonstrate how making FRP school breakfast more accessible increases student participation and can contribute to how decision makers—from legislators to cafeteria and meal service directors—address child food insecurity and nutrition through increased access. This is of particular importance for FRP-eligible students who most benefit. Particularly, as most studies have looked at CEP or BATB in isolation and not considered potential confounders, the deployment of a conditional DiD approach offered a unique contribution to the evidence base around school meal service efficiency, effectiveness, and equity. 

Looking ahead to the likely expiration of universal school meal flexibilities in place due to the COVID-19 pandemic, schools will soon seek other means and mechanisms for extending or expanding student meal access and participation. Notably, the USD 1.7 trillion “Build Back Better” social welfare spending proposal, which at the time of this study passed the U.S. House of Representatives and remains under consideration by the U.S. Senate, proposes lowering the CEP school eligibility threshold from 40% to 25% ISP. Our findings suggest whether through existing mechanisms or potential future expansions of CEP or universal free meals at the state and federal level CEP adoption or universal free meals is the first critical step for expanding meal access. CEP alone, however, as suggested by our findings, is not enough to increase meal participation, and schools should incorporate BATB approaches in tandem with the administrative updates. 

In conclusion, by comprehensively examining the different program and policy approaches schools can take to increase school meal participation, this study offers meaningful insights and recommendations for school administrators and policy makers alike who are interested in decreasing childhood food insecurity and increasing nutrition through the School Breakfast Program and National School Lunch Programs.

## Figures and Tables

**Table 1:** Summary statistics.

	Percent/Mean
School Community Eligibility Provision (CEP) Participation (%) ^1^	
2016–2017	27.4% (42.3%)
2017–2018	30.0% (51.9%)
2018–2019	37.0% (56.8%)
2019–2020	38.1% (60.4%)
School Breakfast after the Bell (BATB Utilization (%)	
2016–2017	
2017–2018	36.3%
2018–2019	44.4%
2019–2020	46.3%
Student Free and Reduced-Price (FRP) Breakfast Participation (% of eligible students)	
2016–2017	48.8%
2017–2018	49.0%
2018–2019	49.4%
2019–2020	51.1%
Control Variables	
Students (K to 12, # ^2^)	413.5
White, non-Hispanic (%)	72.6%
Rural	62.0%
Low access to food	41.8%
School type	
Public	96.1%
Public charter	3.2%
Non-public	0.7%

^1^ Participation rates of CEP-eligible schools are provided in parentheses. ^2^ Number of enrolled students in each school.

**Table 2:** Conditional DiD results.

	BATB
Temporal Mean Difference	
Treatment	0.008
Controls	−0.006
Diff-in-Diff	0.014 * (0.006)
Observations	
Treatment	753
Comparison	373

* *p* < 0.05; Abadie and Imbens robust standard errors in parentheses.

**Table 3:** Fixed Effects Difference-in-Differences.

	BATB
Treated	-
Post	−0.035 *** (0.006)
Treated × Post	0.015 ** (0.005)
School year	
2017–2018	−0.000 (0.002)
2018–2019	0.029 *** (0.005)
2019–2020	0.068 *** (0.002)
Cons.	0.472 *** (0.002)
Observations	4165
sigma u	0.159
sigma e	0.063
rho	0.866

** *p* < 0.01, *** *p* < 0.001; Robust standard errors in parentheses.

## Data Availability

Data for this study were obtained from the Missouri Department of Elementary and Secondary Education available online at https://dese.mo.gov/school-data and https://dese.mo.gov/financial-admin-services/food-nutrition-services/statistics accessed 1 June 2021 and through supplemental e-mail request.
